# Association between physical activity and the prevalence of metabolic syndrome: from the Korean National Health and Nutrition Examination Survey, 1999–2012

**DOI:** 10.1186/s40064-016-3514-5

**Published:** 2016-10-25

**Authors:** Junga Lee, Yoonmyung Kim, Justin Y. Jeon

**Affiliations:** 1 Department of Sport and Leisure Studies, Yonsei University, Seoul, South Korea; 2 Exercise Medicine Center for Diabetes and Cancer Patients, Yonsei University, Seoul, South Korea

**Keywords:** Physical activity, Metabolic syndrome, Korean

## Abstract

The purpose of this study was to determine favorable physical activities, by intensity, type, and frequency that would serve to minimize the prevalence of metabolic syndrome. A total of 24,178 individuals participated in the Korean National Health and Nutrition Examination Survey that investigated the risk factors for metabolic syndrome and physical activity including the physical activity type (vigorous and moderate activity, walking, strength, and flexibility) and the frequency (numbers of days per weeks where physical activity lasted at least 10 min per session). Complex-samples analysis, descriptive statistics, Pearson’s Chi-square test, and multivariate logistic regression analyses were conducted to analyze the data. The results confirmed that physical activity had a favorable effect on reducing the occurrence of metabolic syndrome. The lowest prevalence of metabolic syndrome was observed when vigorous physical activity was conducted six times per week (OR 0.65, 95% CI 0.45–0.94). The associated beneficial effects included improvements in the risk factors of metabolic syndrome, depending on the specific physical activity type and frequency. Patients diagnosed, as having metabolic syndrome should be aware of the associated underling risk factors in order to determine a targeted physical activity intervention.

**Electronic supplementary material:**

The online version of this article (doi:10.1186/s40064-016-3514-5) contains supplementary material, which is available to authorized users.

## Background

Metabolic syndrome is an cluster of risk of diabetes and cardiovascular disease risk factor (Gami et al. 2007). According to the National Health and Nutrition Examination Survey (NHANES) and as defined by the National Cholesterol Education Program Adult Treatment Panel (NCEP-ATP III), the age adjusted prevalence of metabolic syndrome has increased from 29.2% (from the 1988 to 1994 study) to 34.2% (from the 1999 to 2006 study) in the United States (Mozumdar and Liguori 2011). The Korean National Health and Nutrition Examination Survey (KNHANES), as defined by NCEP-ATP III, estimated that the age-adjusted prevalence of metabolic syndrome has increased from 24.9% in 1998 to 31.5% in 2010 (Lim et al. 2011). The prevalence of metabolic syndrome has gradually increased throughout the recent decade across the world.

Metabolic syndrome is defined by the presence of three or more of the following four risk factors: dyslipidemia, hyperglycemia, abdominal obesity, and hypertension (Grundy et al. 2005). These risk factors have been linked to such serious health problems as type 2 diabetes and cardiovascular disease. It is well known that increased physical activity has been associated with lower prevalence of metabolic syndrome, type 2 diabetes, and cardiovascular disease (LaMonte et al. 2005). Also, cardiorespiratory fitness has been shown to be inversely associated with the prevalence of metabolic syndrome (Hong et al. 2014). In a meta-analysis, He et al. (2014) found that high and moderate levels of leisure time physical activity were respectively associated with a 20 and 5% decreased risk of metabolic syndrome. Another meta-analysis (Huang and Liu 2014) reported that moderate and high levels of leisure time physical activity had an 11 and 42% decreased risk of metabolic syndrome.

Although several studies have identified the association between physical activity and metabolic syndrome (Huang and Liu 2014; Rennie et al. 2003; Hastert et al. 2015; Owlasiuk et al. 2014; Petersen et al. 2014), the beneficial effects toward the prevention of metabolic syndrome, according to type and frequency of physical activity, have not been fully investigated. More detailed research as to the appropriate type and frequency of physical activity is necessary, in order to properly encourage physical activity and to determine the correct level of physical activity participation that will help to prevent and reduce the prevalence of metabolic syndrome. Thus, the purpose of this study was to determine the association between the risk components of metabolic syndrome and the type and frequency of various physical activities. This research further investigated the association between the frequency of physical activity per week and the type of physical activity compared to the prevalence of metabolic syndrome.

## Methods

This study used a cross-sectional design to determine the association between physical activity and prevalence of metabolic syndrome. The original data source was the Korean National Health and Health and Nutrition Examination Survey (KNHANES), which is a national representative statistical study on health and nutrition using a stratified, multistage probability sampling design for the selection of household units, and was conducted by the Korean Ministry of Health and Welfare in 1999, 2010, 2011, and 2012.

A total of 24,178 individuals, from 19 to 60 years of age, participated in health interviews and examinations by trained data collectors. Body weight was measured to the nearest 0.1 kg on a balance scale, and height was measured to the nearest 0.1 cm. Based on the weight and height, the body mass index (BMI) was calculated as the weight (kilograms) divided by the height in meters squared. Waist circumference was measured at the most narrow point midway between the lower border of the rib cage and the iliac crest, without placing pressure on the skin (WHO 1987). Using a measuring tape, the waist measurements were taken immediately following normal expiration. The participant’s blood pressure was taken twice, after having remained quietly in a seated position, for about 10 min. An average of the first and second blood pressure measurements was used. Blood samples were drawn from participants after fasting for 8 h or more, to measure triglycerides, HDL cholesterol, and serum glucose concentrations.

The determination of metabolic syndrome followed the metabolic syndrome criteria as used by the (American Heart Association, National Heart Lung and Blood Institute for Asians) (Grundy et al. 2005). These criteria were: triglyceride (≥150 mg/dL), HDL cholesterol (<40 mg/dL for male and <50 mg/dL for female), serum glucose concentration (100 mg/dL for both male and female), and blood pressure (≥130/85 mmHg for both male and female). The criteria for waist circumference (≥90 cm for male and ≥85 cm for female) followed the metabolic syndrome criteria of the Korean Society for the Study of Obesity (Lee et al. 2006).

This study used a physical activity questionnaire that was a version of the International Physical Activity Questionnaire-Short Form (IPAQ-Short) and added two more questions about activities of strength and flexibility. This questionnaire allowed for the collection of data covering vigorous, moderate, and walking physical activities, to include activities related to strength and flexibility. This physical activity questionnaire is presented in Additional file 1: Table S1. This study used five answers from IPAQ, which for that week of the survey identified the number of days the participants did vigorous physical activity, moderate physical activity, walking, strength, and flexibility, for at least 10 min at a time.

**Table 1:** Baseline characteristics of the study population

	Total N = 24,178	MS yes N = 4545 18.8%	MS no N = 19,633 81.2%	Male N = 10,394	MS yes N = 2401 23.1%	MS no N = 7993 76.9%	Female N = 13,784	MS yes N = 1957 14.2%	MS no N = 11,827 85.8%
Age (year)	40.86 ± 0.13	47.71 ± 0.21	39.28 ± 0.14	40.61 ± 0.17	46.06 ± 0.26	38.96 ± 0.19	41.13 ± 0.15	50.53 ± 0.28	39.58 ± 0.15
Height (cm)	165.1.0 ± 0.08	165.54 ± 0.17	165.1.0 ± 0.09	171.45 ± 0.08	170.84 ± 0.15	171.64 ± 0.10	158.46 ± 0.07	156.52 ± 0.15	158.78 ± 0.07
Weight (kg)	64.73 ± 0.11	73.27 ± 0.23	62.76 ± 0.11	71.24 ± 0.14	77.51 ± 0.29	69.36 ± 0.15	57.93 ± 0.11	66.03 ± 0.31	56.59 ± 0.10
BMI (kg/m^2^)	23.66 ± 0.03	26.64 ± 0.06	22.97 ± 0.3	24.20 ± 0.04	26.49 ± 0.08	23.51 ± 0.04	23.09 ± 0.04	26.91 ± 0.11	22.46 ± 0.04
WC (cm)	80.64 ± 0.11	90.27 ± 0.17	78.42 ± 0.10	84.08 ± 0.13	91.36 ± 0.21	81.89 ± 0.13	77.05 ± 0.13	88.40 ± 0.27	75.17 ± 0.13
SBP (mmHg)	115.72 ± 0.17	132.12 ± 0.31	111.94 ± 0.14	119.11 ± 0.20	132.06 ± 0.36	115.21 ± 0.18	112.18 ± 0.19	132.22 ± 0.48	108.87 ± 0.17
DBP (mmHg)	76.73 ± 0.13	87.66 ± 0.21	74.20 ± 0.13	79.91 ± 0.16	89.50 ± 0.25	77.02 ± 0.16	73.40 ± 0.14	84.52 ± 0.29	71.57 ± 0.13
Glucose (mmol/L)	95.75 ± 0.17	110.97 ± 0.55	92.24 ± 0.14	97.68 ± 0.25	110.43 ± 0.67	93.84 ± 0.22	93.74 ± 0.21	111.89 ± 0.92	90.74 ± 0.16
HbA1c (%)	5.78 ± 0.02	6.42 ± 0.04	5.60 ± 0.01	5.84 ± 0.02	6.30 ± 0.04	5.66 ± 0.02	5.73 ± 0.02	6.62 ± 0.06	5.53 ± 0.01
TC (mg/dL)	186.60 ± 0.33	200.17 ± 0.68	183.46 ± 0.35	187.32 ± 0.48	198.54 ± 0.84	183.95 ± 0.52	185.84 ± 0.40	202.94 ± 1.11	183.01 ± 0.39
TG (mg/dL)	133.31 ± 1.00	234.63 ± 3.21	109.91 ± 0.75	158.73 ± 1.59	256.29 ± 4.08	129.36 ± 1.39	106.74 ± 0.98	197.70 ± 4.63	91.71 ± 0.60
HDL-C (mg/dL)	52.69 ± 0.12	44.53 ± 0.20	54.57 ± 0.13	49.38 ± 0.14	43.54 ± 0.24	51.14 ± 0.18	56.15 ± 0.15	46.23 ± 0.29	57.79 ± 0.15
LDL-C (mg/dL)	112.64 ± 0.53	119.50 ± 1.15	110.52 ± 0.58	113.92 ± 0.72	117.66 ± 1.42	112.44 ± 0.81	110.88 ± 0.75	123.66 ± 1.95	108.22 ± 0.78
Postmenopausal (N)	–	–	–	–	–	–	5604	876	4728

Values are mean ± SD. *MD* metabolic syndrome, *HR* heart rate, *BMI* body mass index, *WC* waist circumference, *SBP* systolic blood pressure, *DBP* diastolic blood pressure, *HbA1c* hemoglobin A1c, *TC* total cholesterol, *TG* triglyceride, *HDL-C* high density lipoprotein cholesterol, *LDL-C* low density lipoprotein cholesterol

The options for participation level were: (1) I did not do any physical activity, (2) 1 day per week, (3) 2 days per week, (4) 3 days per week, (5) 4 days per week, (6) 5 days per week, (7) 6 days per week, and (8) every day. Also, for the categories of strengthening and stretching, participants had to select one option that best described their level of participation during the week. The options were: (1) I did not do any physical activity, (2) 1 day per week, (3) 2 days per week, (4) 3 days per week, (5) 5 days per week, and (6) more than 5 days per week.

### Data analysis

This study calculated the frequency of physical activities by type, and determined the arithmetic means of the participants’ general characteristics, including weight, height, BMI, waist circumstance, fasting glucose, triglycerides, HDL cholesterol, and blood pressure. A Pearson’s Chi-square test was performed to investigate the association between the type and frequency of physical activity, and the frequency of occurrence of metabolic syndrome. Multivariable logistic regression analysis was conducted to examine the odds ratios (OR) and 95% confidence intervals (CIs) regarding the association between type and frequency of physical activity and the prevalence of metabolic syndrome. Statistical significance was defined as *p* < 0.05. All statistical analyses were performed using the Statistical Package for the Social Sciences 20.0 (SPSS, Chicago, IL, USA).

## Results

A total of 24,178 participants completed this study (4545 had metabolic syndrome). The characteristics of the study population are presented in Table 1.

### Association between each component of metabolic syndrome and physical activity according to type and frequency of physical activity

Overall, more physically active participants showed lower prevalence of metabolic syndrome (Table 2). Participants who had at least one per week or more per week of physical activity showed significantly lower levels for their components of metabolic syndrome in the categories of fasting glucose, HDL cholesterol, and waist circumference, compared with participants who did not participant in any physical activity. The risk components of metabolic syndrome were lower for the subjects participating in vigorous physical activity, moderate physical activity, walking, strength, and flexibility, than for the subjects who did not participated in physical activity.

**Table 2:** Means of metabolic syndrome components according to type and frequency of physical activity

Physical activity		Fasting blood glucose (mg/dL)	Triglycerides (mg/dL)	HDL cholesterol (mg/dL)	Waist circumference (cm)	Systolic blood pressure (mmHg)	Diastolic blood pressure (mmHg)
*Vigorous*							
0/week	Total	96.16 ± 0.21 (95.75, 96.57)	134.04 ± 1.16 (131.77, 136.32)	52.38 ± 0.13 (52.12, 52.64)	80.87 ± 0.07 (80.72, 81.01)	113.61 ± 0.22 (113.17, 114.04)	74.76 ± 0.18 (74.41, 75.11)
	M	98.33 ± 0.34 (97.66, 99.01)	159.14 ± 1.88 (155.44, 162.83)	48.90 ± 0.19 (48.53, 49.28)	84.42 ± 0.09 (84.25, 84.59)	119.12 ± 0.25 (118.64, 119.61)	79.74 ± 0.19 (79.37, 80.11)
	F	93.95 ± 0.24 (93.49, 94.41)	108.44 ± 1.13 (106.22, 110.66)	55.87 ± 0.16 (55.57, 56.17)	77.26 ± 0.09 (77.09, 77.43)	112.34 ± 0.19 (111.97, 112.71)	73.37 ± 0.14 (73.09, 73.65)
1/week	Total	95.07 ± 0.39 (94.31, 95.83)*	135.15 ± 2.71 (129.82, 140.47)	53.00 ± 0.28 (52.45, 53.55)*	80.44 ± 0.11 (80.22, 80.66)**	113.85 ± 0.39 (113.08, 114.61)	75.49 ± 0.32 (74.86, 76.11)*
	M	96.97 ± 0.44 (96.12, 97.83)*	164.57 ± 3.79 (157.14, 172.00)	49.35 ± 0.33 (48.70, 50.00)	84.08 ± 0.13 (83.83, 84.32)**	119.08 ± 0.42 (118.26, 119.89)	80.36 ± 0.34 (79.69, 81.03)
	F	93.67 ± 0.64 (92.41, 94.92)	102.22 ± 2.50 (97.31, 107.13)*	56.96 ± 0.44 (56.09, 57.83)	76.65 ± 0.18 (76.29, 77.00)**	111.44 ± 0.49 (110.49, 112.40)	73.72 ± 0.36 (73.01, 74.43)
2/week	Total	95.19 ± 0.41 (94.38, 96.00)*	140.50 ± 4.05 (132.56, 148.44)	52.70 ± 0.31 (52.10, 53.31)**	80.24 ± 0.13 (79.98, 80.49)**	112.91 ± 0.42 (112.08, 113.74)	74.78 ± 0.29 (74.21, 75.35)
	M	97.06 ± 0.53 (96.02, 98.10)*	167.92 ± 6.15 (155.86, 179.99)	49.79 ± 0.40 (49.00, 50.58)**	83.88 ± 0.16 (83.56, 84.19)**	118.88 ± 0.47 (117.95, 119.80)	80.23 ± 0.36 (79.53, 80.93)
	F	93.46 ± 0.63 (92.22, 94.69)	111.72 ± 3.27 (105.30, 118.13)	55.22 ± 0.476 (54.28, 56.15)*	76.46 ± 0.18 (76.12, 76.81)**	111.35 ± 0.55 (110.28, 111.43)	73.03 ± 0.34 (72.37, 73.69)
3/week	Total	95.48 ± 0.52 (94.45, 96.51)*	128.65 ± 3.35 (122.07, 135.23)	53.53 ± 0.33 (52.88, 54.19)*	79.96 ± 0.14 (79.68, 80.24)**	114.13 ± 0.48 (113.18, 115.07)	75.55 ± 0.36 (74.84, 76.27)*
	M	98.00 ± 0.78 (96.47, 99.52)	155.39 ± 5.30 (144.99, 165.79)*	50.00 ± 0.45 (49.11, 50.88)	83.37 ± 0.17 (83.04, 83.70)**	119.64 ± 0.56 (118.54, 120.75)	80.50 ± 0.45 (79.62, 81.39)
	F	92.65 ± 0.64 (91.40, 93.91)*	101.99 ± 3.06 (95.99, 107.99)*	57.47 ± 0.90 (55.71, 59.23)*	76.57 ± 0.22 (76.14, 77.00)**	111.80 ± 0.58 (110.66, 112.94)	73.51 ± 0.38 (72.76, 74.26)
4/week	Total	94.22 ± 0.60 (93.04, 95.40)	120.85 ± 4.30 (112.42, 129.29)	53.60 ± 0.56 (52.50, 54.70)**	79.95 ± 0.19 (79.58, 80.32)**	114.65 ± 0.74 (113.19, 116.11)	75.25 ± 0.54 (74.19, 76.30)
	M	95.46 ± 0.82 (93.85, 97.06)**	142.14 ± 6.67 (129.05, 155.24)*	49.91 ± 0.71 (48.52, 51.31)*	83.57 ± 0.26 (83.07, 84.07)**	119.22 ± 0.73 (117.79, 120.64)	79.64 ± 0.58 (78.49, 80.79)
	F	93.53 ± 0.96 (91.64, 95.41)	100.87 ± 4.07 (92.89, 108.85)	58.71 ± 0.83 (57.09, 60.33)**	76.21 ± 0.28 (75.67, 76.75)**	113.25 ± 0.97 (111.35, 115.16)	74.17 ± 0.68 (72.83, 75.51)
5/week	Total	94.43 ± 0.78 (92.89, 95.97)*	122.42 ± 4.45 (113.68, 131.16)*	54.61 ± 0.53 (53.57, 55.65)**	79.70 ± 0.20 (79.32, 80.09)**	114.46 ± 0.65 (113.19, 115.73)	75.46 ± 0.52 (74.45, 76.48)
	M	95.93 ± 1.04 (93.89, 97.97)*	150.64 ± 6.59 (137.71, 163.57)	50.72 ± 0.65 (49.44, 51.99)	83.13 ± 0.29 (82.57, 83.70)**	120.10 ± 0.83 (118.47, 121.73)	80.13 ± 0.64 (78.87, 81.39)
	F	93.24 ± 1.20 (90.88, 95.60)	93.75 ± 4.45 (85.02, 102.49)*	58.71 ± 0.83 (57.09, 60.96)**	76.28 ± 0.34 (75.81, 76.74)**	112.40 ± 0.76 (110.91, 113.89)	73.58 ± 0.50 (72.60, 74.55)
6/week	Total	94.41 ± 0.80 (92.23, 96.59)*	121.54 ± 6.56 (108.67, 134.42)*	54.47 ± 0.76 (52.99, 55.96)**	79.22 ± 0.26 (78.70, 79.74)**	114.07 ± 0.99 (112.12, 116.01)	75.39 ± 0.74 (73.93, 76.84)
	M	97.58 ± 1.50 (94.62, 100.53)	148.22 ± 9.07 (130.42, 166.03)	50.83 ± 0.92 (49.02, 52.64)**	82.65 ± 0.32 (82.02, 83.29)**	118.50 ± 0.91 (116.71, 120.29)	79.24 ± 0.83 (77.60, 80.87)
	F	89.57 ± 0.90 (87.81, 91.33)**	94.12 ± 6.08 (82.18, 106.06)*	58.42 ± 1.30 (55.88, 60.96)	75.93 ± 0.43 (75.08, 76.77)**	112.16 ± 1.27 (109.68, 113.65)	73.03 ± 0.85 (71.37, 74.69)
Everyday	Total	94.76 ± 0.80 (93.20, 96.33)	111.83 ± 4.69 (102.62, 121.04)**	54.27 ± 0.61 (53.08, 55.46)*	80.34 ± 0.20 (79.94, 80.74)*	113.34 ± 0.77 (111.83, 114.86)	74.44 ± 0.59 (73.28, 75.60)
	M	96.21 ± 0.96 (94.32, 98.10)*	133.07 ± 7.35 (118.65, 147.49)**	51.38 ± 0.75 (49.91, 52.86)*	83.66 ± 0.26 (83.15, 84.17)**	117.62 ± 0.282(116.00, 119.24)	78.33 ± 0.60 (77.15, 79.52)*
	F	93.72 ± 0.1.41 (90.95, 96.50)*	94.74 ± 4.41 (86.08, 103.40)**	56.86 ± 0.95 (55.00, 58.71)	77.14 ± 0.30 (76.55, 77.72)	111.70 ± 1.09 (109.56, 112.84)	73.37 ± 0.74 (71.92, 74.81)
*P* for trend	Total	<0.01	<0.001	<0.001	<0.001	0.26	0.10
	M	<0.01		<0.001	<0.001	0.55	0.06
	F	<0.001	<0.01	<0.001	<0.001	0.30	0.82
*Moderate*							
0/week	Total	95.26 ± 0.38 (94.51, 96.01)	133.80 ± 1.12 (131.61, 135.99)	52.34 ± 0.14 (52.07, 52.61)	80.81 ± 0.07 (80.66, 81.96)	115.77 ± 0.18 (115.42, 116.12)	76.64 ± 0.14 (76.38, 76.91)
	M	97.90 ± 0.33 (97.26, 98.55)	159.19 ± 1.98 (155.53, 163.05)	48.87 ± 0.20 (48.48, 49.27)	84.37 ± 0.09 (84.19, 84.54)	119.24 ± 0.25 (118.75, 119.73)	79.82 ± 0.19 (79.45, 80.20)
	F	94.22 ± 0.25 (93.73, 94.72)	107.83 ± 0.91 (106.05, 109.61)	55.82 ± 0.17 (55.50, 56.15)	77.20 ± 0.09 (77.02, 77.37)	112.33 ± 0.20 (111.93, 112.72)	73.44 ± 0.15 (73.15, 73.74)
1/week	Total	95.80 ± 0.54 (94.75, 96.86)	135.74 ± 2.94 (129.98, 141.50)	52.65 ± 0.27 (52.12, 53.18)**	80.51 ± 0.11 (80.29, 80.73)*	115.69 ± 0.36 (115.00, 116.39)	77.00 ± 0.29 (76.44, 77.57)
	M	97.75 ± 0.51 (96.75, 98.76)	164.01 ± 4.41 (155.36, 172.66)	49.46 ± 0.35 (48.78, 50.14)	84.15 ± 0.13 (83.89, 84.40)	119.05 ± 0.46 (118.15, 119.96)	80.33 ± 0.38 (79.58, 81.08)
	F	92.71 ± 0.48 (91.77, 93.65)**	105.61 ± 2.25 (101.19, 110.02)	55.71 ± 0.40 (54.92, 56.50)	76.72 ± 0.17 (76.38, 77.06)**	112.30 ± 0.49 (111.33, 113.26)	73.54 ± 0.35 (72.86, 74.23)
2/week	Total	96.09 ± 0.55 (95.02, 97.16)	136.11 ± 3.26 (129.71, 142.50)	53.40 ± 0.30 (52.80, 53.99)*	80.34 ± 0.12 (80.10, 80.58)**	114.98 ± 0.33 (114.32, 115.63)	76.67 ± 0.26 (76.16, 77.17)
	M	96.96 ± 0.57 (95.84, 98.08)	162.38 ± 4.86 (152.86, 171.91)**	50.05 ± 0.39 (49.28, 50.81)**	83.77 ± 0.15 (83.47, 84.07)**	118.41 ± 0.44 (117.55, 119.28)	80.15 ± 0.34 (79.49, 80.82)
	F	93.73 ± 0.64 (92.47, 94.99)	109.51 ± 3.85 (101.95, 117.08)*	56.75 ± 0.44 (55.88, 57.61)*	76.88 ± 0.16 (76.56, 77.20)	111.53 ± 0.48 (110.59, 112.47)	73.07 ± 0.34 (72.40, 73.75)
3/week	Total	96.08 ± 0.45 (95.20, 96.96)	136.63 ± 4.41 (127.98, 145.28)	53.29 ± 0.32 (52.65, 53.92)**	80.13 ± 0.13 (79.87, 80.39)**	115.57 ± 0.41 (114.76, 116.38)	76.90 ± 0.31 (76.30, 77.50)
	M	97.72 ± 0.81 (96.14, 99.30)	161.46 ± 5.28 (151.10, 171.81)	49.64 ± 0.44 (48.78, 50.50)	83.58 ± 0.17 (83.25, 83.91)**	119.40 ± 0.58 (118.26, 120.53)	80.21 ± 0.45 (79.33, 81.09)
	F	93.41 ± 0.81 (91.83, 95.00)	111.38 ± 7.44 (96.79, 125.95)*	56.98 ± 0.48 (56.04, 57.91)*	76.64 ± 0.19 (76.27, 77.01)**	111.83 ± 0.51 (110.82, 112.84)	73.62 ± 0.33 (72.97, 74.28)
4/week	Total	95.53 ± 0.52 (94.52, 96.54)	119.13 ± 4.53 (110.23, 128.02)*	54.24 ± 0.49 (53.29, 55.20)*	80.39 ± 0.19 (80.03, 80.76)*	116.05 ± 0.60 (114.87, 117.22)	76.63 ± 0.44 (75.78, 77.48)
	M	97.15 ± 0.148 (94.25, 100.06)	142.84 ± 7.31 (128.49, 157.20)*	50.54 ± 0.66 (49.25, 51.82)*	84.18 ± 0.25 (83.68, 84.67)	120.47 ± 0.79 (118.42, 119.99)	80.26 ± 0.60 (79.37, 80.33)
	F	92.31 ± 1.04 (90.26, 94.35)	95.72 ± 3.69 (88.48, 102.95)**	58.13 ± 0.76 (56.65, 59.61)**	76.43 ± 0.26 (75.92, 76.94)**	111.09 ± 0.87 (109.38, 112.80)	72.74 ± 0.58 (71.61, 73.88)
5/week	Total	95.66 ± 0.43 (94.81, 96.51)	128.77 ± 4.63 (119.69, 137.85)	53.68 ± 0.44 (52.81, 54.55)**	79.75 ± 0.17 (79.41 80.08)**	115.37 ± 0.48 (114.42, 116.32)	76.26 ± 0.34 (75.60, 76.93)
	M	98.60 ± 0.1.46 (95.73, 101.47)	162.89 ± 8.03 (1147.11, 178.68)	49.94 ± 0.65 (48.68, 51.21)	83.18 ± 0.25 (82.69, 83.66)**	118.70 ± 0.66 (117.42, 119.99)	79.35 ± 0.50 (78.37, 80.33)
	F	90.85 ± 0.50 (89.87, 91.84)**	94.31 ± 3.42 (87.60, 101.03)**	57.53 ± 0.60 (56.36, 58.70)**	76.31 ± 0.21 (75.90, 76.71)**	111.74 ± 0.67 (110.42, 113.06)	73.14 ± 0.42 (72.32, 73.95)
6/week	Total	95.27 ± 0.47 (94.35, 96.20)	122.24 ± 5.55 (111.34, 133.14)*	54.20 ± 0.62 (52.99, 55.41)*	79.91 ± 0.21 (79.50, 80.32)**	115.79 ± 0.71 (114.41, 117.17)	76.83 ± 0.53 (75.78, 77.88)
	M	95.55 ± 0.1.04 (93.50, 97.60)*	147.74 ± 8.68 (130.71, 164.77)**	51.18 ± 0.78 (49.66, 52.70)**	82.89 ± 0.25 (82.39, 83.39)**	118.08 ± 0.92 (116.28, 119.89)	79.26 ± 0.74 (77.80, 80.71)
	F	93.54 ± 1.21 (91.16, 95.93)**	97.97 ± 5.77 (86.65, 109.28)	57.01 ± 1.02 (55.01, 59.01)	77.18 ± 0.33 (76.53, 77.83)	114.04 ± 1.02 (112.04, 116.04)	74.85 ± 0.72 (73.45, 76.25)
Everyday	Total	95.77 ± 0.27 (95.24, 96.30)	122.14 ± 2.99 (116.25, 128.02)**	53.13 ± 0.37 (52.40, 53.85)*	80.66 ± 0.16 (80.36, 80.97)	115.58 ± 0.46 (114.67, 116.48)	76.12 ± 0.35 (75.44, 76.81)
	M	97.39 ± 0.93 (95.57, 99.21)	143.92 ± 5.07 (133.97, 153.86)**	50.00 ± 0.53 (48.97, 51.04)*	83.91 ± 0.20 (83.52, 84.31)*	118.79 ± 0.60 (117.60, 119.97)	79.28 ± 0.49 (78.32, 80.23)
	F	93.11 ± 0.71 (91.72, 94.50)	103.62 ± 2.76 (98.21, 109.02)	56.24 ± 0.49 (55.27, 57.21)	77.47 ± 0.22 (77.04, 77.88)	112.04 ± 0.59 (110.89, 113.19)	73.00 ± 0.39 (72.23, 73.77)
*P* for trend	Total	0.84	<0.001	<0.001	<0.001	0.56	0.458
	M	0.46	<0.001	<0.001		0.33	0.47
	F	<0.001	<0.001	<0.001	<0.001	0.28	0.21
*Walking*							
0/week	Total	96.11 ± 0.21 (95.70, 96.51)	135.94 ± 2.12 (131.78, 140.10)	51.89 ± 0.26 (51.39, 52.39)	80.86 ± 0.12 (80.63, 81.09)	115.75 ± 0.30 (115.16, 116.34)	76.64 ± 0.23 (76.18, 76.91)
	M	97.16 ± 0.63 (95.91, 98.40)	158.24 ± 3.63 (151.12, 165.35)	48.52 ± 0.37 (47.79, 49.26)	84.30 ± 0.15 (84.01, 84.59)	118.61 ± 0.44 (117.74, 119.48)	79.58 ± 0.35 (78.91, 80.26)
	F	93.37 ± 0.43 (92.52, 94.22)	113.40 ± 2.18 (109.11, 117.68)	55.29 ± 0.32 (54.65, 55.92)	77.36 ± 0.15 (77.07, 77.66)	112.89 ± 0.43 (112.06, 113.73)	73.67 ± 0.30 (73.09, 74.26)
1/week	Total	95.34 ± 0.37 (94.61, 96.08)**	139.31 ± 2.91 (133.61, 145.01)	52.35 ± 0.35 (51.66, 53.04)	81.07 ± 0.14 (80.80, 81.35)	115.44 ± 0.40 (114.65, 116.23)	77.00 ± 0.31 (76.40, 77.60)
	M	97.59 ± 0.78 (96.06, 99.12)	167.83 ± 4.86 (158.28, 177.37)	48.92 ± 0.47 (48.00, 49.83)	84.66 ± 0.19 (84.29, 85.03)	119.19 ± 0.61 (117.99, 120.38)	80.77 ± 0.46 (79.87, 81.68)*
	F	93.93 ± 0.73 (92.49, 95.36)	108.32 ± 2.38 (103.66, 112.98)	55.82 ± 0.51 (54.82, 56.82)	77.37 ± 0.18 (77.02, 77.71)	111.79 ± 0.51 (110.78, 112.80)	73.10 ± 0.37 (72.38, 73.83)
2/week	Total	95.29 ± 0.44 (94.42, 96.16)**	135.73 ± 2.50 (130.82, 140.63)	51.92 ± 0.25 (51.42, 52.41)	80.76 ± 0.11 (80.54, 80.99)	114.73 ± 0.35 (114.04, 115.41)*	76.57 ± 0.25 (76.08, 77.06)
	M	99.20 ± 1.00 (97.24, 101.16)	162.33 ± 4.46 (153.58, 171.09)	48.47 ± 0.37 (47.75, 49.18)	84.32 ± 0.15 (84.03, 84.61)	118.57 ± 0.53 (117.54, 119.60)	80.23 ± 0.39 (79.47, 80.99)
	F	92.82 ± 0.44 (91.96, 93.69)	107.93 ± 2.33 (103.36, 112.51)	55.36 ± 0.35 (54.67, 56.04)*	77.13 ± 0.16 (76.82, 77.44)	111.08 ± 0.40 (110.30, 111.87)	72.94 ± 0.28 (72.38, 73.49)
3/week	Total	95.68 ± 0.59 (94.52, 96.84)**	134.78 ± 2.18 (130.49, 139.06)	52.50 ± 0.25 (52.00, 53.00)	80.74 ± 0.11 (80.53, 80.95)	115.41 ± 0.32 (114.78, 116.04)	76.83 ± 0.24 (76.36, 77.29)
	M	97.83 ± 0.58 (96.70, 98.96)	160.67 ± 4.13 (152.57, 168.77)	48.77 ± 0.36 (48.06, 49.48)	84.16 ± 0.15 (83.87, 84.46)	118.87 ± 0.46 (117.97, 119.78)	80.10 ± 0.36 (79.40, 80.80)
	F	94.23 ± 0.63 (92.99, 95.46)	107.98 ± 1.94 (104.17, 111.79)**	56.20 ± 0.34 (55.54, 56.87)**	77.23 ± 0.14 (76.96, 77.51)	112.02 ± 0.39 (111.27, 112.78)	73.53 ± 0.27 (73.00, 74.07)
4/week	Total	94.72 ± 0.96 (92.83, 96.60)**	139.40 ± 3.67 (132.20, 146.60)	53.23 ± 0.35 (52.54, 53.92)*	80.67 ± 0.14 (80.41, 80.94)	115.46 ± 0.42 (114.63, 116.29)	76.71 ± 0.30 (76.12, 77.31)
	M	97.50 ± 0.80 (95.93, 99.08)	169.84 ± 7.19 (155.73, 183.94)	49.61 ± 0.500 (48.63, 50.59)	83.23 ± 0.19 (83.85, 84.60)	118.93 ± 0.62 (117.72, 120.14)	79.93 ± 0.45 (79.05, 80.82)
	F	93.52 ± 0.63 (92.30, 94.75)	108.34 ± 2.68 (103.08, 113.61)**	56.85 ± 0.45 (55.96, 58.07)**	77.04 ± 0.18 (76.70, 77.39)	112.04 ± 0.53 (110.99, 113.09)	73.44 ± 0.37 (72.71, 74.16)
5/week	Total	94.78 ± 0.84 (93.14, 96.42)**	127.72 ± 2.38 (123.05, 132.39)*	53.16 ± 0.28 (52.61, 53.71)**	80.28 ± 0.12 (80.04 80.52)**	115.97 ± 0.33 (115.32, 116.62)	77.00 ± 0.24 (76.53, 77.47)
	M	97.94 ± 0.71 (96.54, 99.34)	155.49 ± 4.37 (146.91, 164.06)	48.82 ± 0.40 (48.05, 49.60)	83.94 ± 0.18 (83.60, 84.29)	119.50 ± 0.52 (118.49, 120.51)	80.41 ± 0.39 (79.64, 81.18)
	F	93.36 ± 0.49 (92.39, 94.32)	100.32 ± 2.309 (95.78, 102.85)	57.33 ± 0.38 (56.59, 58.07)**	76.60 ± 0.15 (76.31, 76.89)	112.48 ± 0.40 (111.71, 113.26)	73.63 ± 0.26 (73.12, 74.14)
6/week	Total	94.37 ± 0.79 (92.82, 95.91)**	118.59 ± 2.57 (113.55, 123.63)*	53.76 ± 0.39 (53.00, 54.53)**	80.08 ± 0.14 (79.81, 80.52)**	115.41 ± 0.50 (114.43, 116.38)	76.52 ± 0.36 (75.82, 77.23)
	M	97.09 ± 0.75 (95.62, 98.56)	137.38 ± 4.32 (128.90, 145.86)**	50.53 ± 0.58 (49.38, 51.67)**	83.50 ± 0.20 (83.10, 83.90)**	118.31 ± 0.69 (116.95, 119.76)	79.25 ± 0.55 (78.18, 80.32)
	F	93.34 ± 0.58 (92.19, 94.48)	99.63 ± 2.61 (94.51, 104.76)	57.00 ± 0.49 (56.03, 57.96)**	76.62 ± 0.18 (76.26, 76.98)**	112.58 ± 0.62 (111.36, 113.79)	73.82 ± 0.42 (73.00, 74.64)
Everyday	Total	95.17 ± 0.59 (94.01, 96.32)*	131.23 ± 2.00 (127.32, 135.15)	53.13 ± 0.17 (52.80, 53.47)**	80.44 ± 0.08 (80.28, 80.97)**	116.06 ± 0.21 (115.65, 116.47)	76.48 ± 0.17 (76.15, 76.81)
	M	97.47 ± 0.39 (96.70, 98.24)	157.66 ± 2.82 (152.11, 163.20)	50.16 ± 0.23 (49.71, 50.60)**	83.89 ± 0.10 (83.69, 84.08)*	119.58 ± 0.28 (119.03, 120.13)	79.67 ± 0.23 (79.22, 80.11)
	F	94.28 ± 0.38 (93.54, 95.03)	106.06 ± 2.19 (101.76, 110.36)	56.04 ± 0.24 (55.58, 56.50)	76.99 ± 0.11 (76.78, 77.19)*	112.29 ± 0.26 (111.77, 112.80)	73.28 ± 0.20 (72.89, 73.67)
*P* for trend	Total	0.80	<0.001	<0.001	<0.001	<0.005	0.515
	M	0.30	<0.001		<0.001	0.33	0.20
	F		<0.005	<0.001	<0.001	0.07	0.42
*Strength*							
0/week	Total	96.00 ± 0.19 (95.63, 96.37)	135.25 ± 1.06 (133.17, 137.32)	52.39 ± 1.13 (52.12, 52.65)	80.86 ± 0.07 (80.72, 81.00)	115.67 ± 0.17 (115.34, 116.00)	76.71 ± 0.13 (76.44, 76.97)
	M	97.98 ± 0.32 (97.35, 98.61)	162.84 ± 1.93 (159.04, 166.63)	48.87 ± 0.18 (48.51, 49.24)	84.46 ± 0.08 (84.30, 84.63)	119.08 ± 0.24 (118.61, 119.55)	79.98 ± 0.20 (79.60, 80.37)
	F	93.94 ± 0.21 (93.54, 94.35)	107.48 ± 0.83 (105.85, 109.11)	55.91 ± 0.15 (55.62, 56.21)	77.20 ± 0.08 (77.03, 77.36)	112.27 ± 0.18 (111.93, 112.62)	73.43 ± 0.13 (73.16, 73.69)
1/week	Total	95.34 ± 0.56 (94.23, 96.44)	133.91 ± 3.37 (127.29, 140.52)	52.77 ± 0.35 (52.07, 53.46)	80.28 ± 0.14 (80.01, 80.54)**	115.29 ± 0.41 (114.49, 116.08)	76.72 ± 0.30 (76.13, 77.31)
	M	97.42 ± 0.77 (95.90, 98.94)	162.42 ± 4.90 (152.81, 172.03)	49.18 ± 0.44 (48.32, 50.04)	83.72 ± 0.16 (83.40, 84.04)**	118.65 ± 0.52 (117.63, 119.68)	80.12 ± 0.40 (79.33, 80.90)
	F	93.32 ± 0.72 (91.90, 94.74)	103.63 ± 3.09 (97.57, 109.69)	56.47 ± 0.58 (55.32, 57.61)	76.93 ± 0.21 (76.52, 77.35)	111.90 ± 0.60 (110.72, 113.09)	73.14 ± 0.42 (72.31, 73.97)
2/week	Total	95.14 ± 0.51 (94.14, 96.14)	130.89 ± 3.56 (123.91, 137.88)	53.00 ± 0.35 (52.32, 53.69)*	80.20 ± 0.14 (79.92, 80.49)**	115.47 ± 0.44 (114.60, 116.33)*	76.35 ± 0.33 (75.70, 76.99)
	M	97.18 ± 0.63 (95.95, 98.40)	157.58 ± 4.95 (147.86, 167.30)	49.34 ± 0.42 (48.52, 50.16)	83.89 ± 0.18 (83.54, 84.24)**	118.94 ± 0.54 (117.88, 120.00)	79.63 ± 0.42 (78.81, 80.45)
	F	93.35 ± 0.72 (91.93, 94.77)	105.77 ± 3.55 (98.80, 112.73)	56.89 ± 0.56 (55.80, 57.98)	76.39 ± 0.20 (76, 76.77)**	111.68 ± 0.69 (110.32, 113.03)	72.98 ± 0.43 (72.15, 73.82)
3/week	Total	94.48 ± 0.49 (93.51, 95.45)**	124.57 ± 4.22 (116.29, 132.84)*	53.83 ± 0.39 (53.07, 54.59)**	79.98 ± 0.14 (79.70, 80.25)**	116.00 ± 0.47 (115.07, 116.92)	77.05 ± 0.32 (76.42, 77.68)
	M	96.99 ± 0.67 (95.67, 98.31)	150.61 ± 5.95 (138.93, 162.30)	50.36 ± 0.47 (49.44, 51.28)**	83.57 ± 0.17 (83.23, 83.90)**	119.35 ± 0.55 (118.27, 120.44)	80.10 ± 0.41 (79.30, 80.90)
	F	91.59 ± 0.59 (90.44, 92.74)**	98.69 ± 3.28 (92.24, 105.13)**	57.32 ± 0.65 (56.05, 58.59)*	76.31 ± 0.23 (75.85, 76.77)**	112.61 ± 0.76 (111.13, 114.09)	74.14 ± 0.48 (73.19, 75.09)
4/week	Total	93.42 ± 0.64 (92.17, 94.68)**	119.29 ± 4.07 (111.30, 127.27)**	54.85 ± 0.51 (53.83, 55.85)**	79.78 ± 0.21 (79.37, 80.19)**	114.93 ± 0.57 (113.81, 116.05)	76.39 ± 0.46 (75.49, 77.29)
	M	95.64 ± 0.65 (94.37, 96.92)	143.55 ± 5.43 (132.90, 154.20)**	51.51 ± 0.60 (50.33, 52.68)**	83.41 ± 0.26 (82.90, 83.93)**	118.87 ± 0.71 (117.48, 120.27)	79.54 ± 0.59 (78.38, 80.71)
	F	91.16 ± 1.46 (88.29, 94.03)	98.63 ± 4.84 (89.13, 108.14)	58.01 ± 0.96 (56.12, 59.90)*	76.05 ± 0.29 (75.47, 76.62)**	110.20 ± 0.89 (108.46, 111.95)*	73.30 ± 0.64 (72.05, 74.55)
5/week	Total	96.07 ± 0.70 (94.71, 97.43)	121.11 ± 5.22 (111.52, 132.01)*	54.43 ± 0.41 (53.62, 55.24)**	79.50 ± 0.15 (79.21 79.80)**	116.18 ± 0.46 (115.29, 117.08)	76.29 ± 0.33 (75.65, 76.93)
	M	98.14 ± 0.70 (96.77, 99.51)	144.85 ± 4.65 (135.73, 153.97)**	50.90 ± 0.48 (49.97, 51.84)**	83.06 ± 0.17 (82.73, 83.39)**	119.85 ± 0.51 (118.84, 120.85)	79.54 ± 0.38 (78.80, 80.29)
	F	94.14 ± 1.82 (90.57, 97.71)	109.32 ± 16.22 (77.49, 141.15)	57.97 ± 0.73 (56.54, 59.41)**	76.04 ± 0.25 (75.54, 76.53)**	111.76 ± 0.75 (110.09, 113.42)	73.07 ± 0.55 (72.00, 74.15)
*P* for trend	Total	<0.001	<0.001	<0.001	<0.001	0.456	0.463
	M	0.04	<0.001		<0.001	0.63	0.76
	F	<0.001	0.05		<0.001	0.23	0.53
*Flexibility*							
0/week	Total	96.09 ± 0.23 (95.63, 96.54)	135.33 ± 1.32 (132.74, 137.92)	52.19 ± 0.15 (51.90, 52.49)	81.00 ± 0.07 (80.85, 81.15)	115.83 ± 0.19 (115.45, 116.20)	76.61 ± 0.15 (76.31, 76.90)
	M	97.75 ± 0.34 (97.09, 98.41)	162.57 ± 2.34 (157.98, 167.16)	48.72 ± 0.21 (48.31, 49.13)	84.47 ± 0.09 (84.31, 84.66)	119.23 ± 0.27 (118.70, 119.77)	79.85 ± 0.21 (79.43, 80.27)
	F	94.47 ± 0.29 (93.90, 95.05)	108.13 ± 1.02 (106.13, 110.13)	55.70 ± 0.19 (55.33, 56.07)	77.46 ± 0.10 (77.28, 77.65)	112.34 ± 0.22 (111.90, 112.77)	73.33 ± 0.16 (73.01, 73.65)
1/week	Total	95.73 ± 0.38 (94.98, 96.47)	135.71 ± 2.52 (130.76, 140.66)	52.85 ± 0.29 (52.28, 53.41)*	80.61 ± 0.20 (80.38, 80.84)**	115.61 ± 0.35 (114.93, 116.30)	76.93 ± 0.26 (76.43, 77.44)
	M	97.83 ± 0.60 (96.66, 99.01)	163.15 ± 4.11 (155.08, 171.22)	49.69 ± 0.40 (48.90, 50.48)*	84.05 ± 0.16 (83.74, 84.36)**	119.04 ± 0.52 (118.02, 120.05)	80.21 ± 0.38 (79.47, 80.95)
	F	93.47 ± 0.53 (92.44, 94.51)	107.13 ± 2.55 (102.12, 112.14)	55.99 ± 0.40 (55.21, 56.77)	77.10 ± 0.16 (76.79, 77.41)*	112.30 ± 0.39 (111.53, 113.08)	73.65 ± 0.32 (73.03, 74.27)
2/week	Total	95.65 ± 0.44 (94.79, 96.51)	134.46 ± 2.38 (129.78, 139.13)	52.84 ± 0.26 (52.33, 53.35)*	80.51 ± 0.12 (80.29, 80.72)**	115.55 ± 3.00 (114.96, 116.14)	76.91 ± 0.22 (76.48, 77.35)
	M	97.68 ± 0.72 (96.28, 99.09)	162.29 ± 4.55 (153.37, 171.22)	49.48 ± 0.35 (48.80, 50.17)	84.04 ± 0.14 (83.76, 84.32)**	119.37 ± 0.45 (118.49, 120.25)	80.50 ± 0.33 (79.85, 81.14)
	F	93.54 ± 0.53 (92.51, 94.57)	106.43 ± 2.18 (102.14, 110.71)	56.22 ± 0.36 (55.51, 56.93)	76.92 ± 0.14 (76.65, 77.19)**	111.83 ± 0.37 (111.11, 112.55)	73.37 ± 0.27 (72.83, 73.91)
3/week	Total	94.96 ± 0.39 (94.19, 95.73)*	128.27 ± 2.04 (124.27, 132.28)*	53.06 ± 0.25 (52.57, 53.55)**	80.32 ± 0.11 (80.10, 80.54)**	115.18 ± 0.35 (114.50, 115.86)	76.49 ± 0.23 (76.04, 76.94)
	M	97.29 ± 0.71 (95.89, 98.69)	152.46 ± 3.92 (144.78, 160.15)*	49.57 ± 0.37 (48.84, 50.29)*	83.92 ± 0.15 (83.62, 84.22)**	118.65 ± 0.52 (117.64, 119.67)	79.71 ± 0.36 (79.01, 80.41)
	F	92.55 ± 0.43 (91.70, 93.40)**	103.38 ± 1.74 (99.97, 106.79)*	56.55 ± 0.33 (55.90, 57.20)*	76.67 ± 0.14 (76.39, 76.94)**	111.80 ± 0.41 (110.99, 112.60)	73.28 ± 0.27 (72.75, 73.81)
4/week	Total	93.25 ± 0.66 (92.95, 95.55)*	125.73 ± 3.00 (119.85, 131.61)*	53.37 ± 0.43 (52.52, 54.21)	79.91 ± 0.16 (79.60, 80.22)**	115.28 ± 0.45 (114.40, 116.17)	76.77 ± 0.36 (76.08, 77.47)
	M	96.17 ± 1.07 (94.07, 98.26)	151.36 ± 5.33 (140.90, 161.81)*	49.83 ± 0.56 (48.74, 50.91)	83.45 ± 0.22 (83.01, 83.89)**	118.55 ± 0.58 (117.41, 119.70)	79.76 ± 0.53 (78.71, 80.81)
	F	92.24 ± 0.72 (90.83, 93.66)**	99.97 ± 2.72 (94.63, 105.31)**	56.94 ± 0.60 (55.76, 58.11)*	76.31 ± 0.20 (75.91, 76.70)**	112.08 ± 0.63 (110.84, 113.32)	73.84 ± 0.40 (73.05, 74.63)
5/week	Total	95.82 ± 0.42 (94.99, 96.66)	128.47 ± 2.73 (123.11, 133.83)*	53.75 ± 0.24 (53.28, 54.22)**	79.95 ± 0.11 (79.75 80.16)**	115.78 ± 0.29 (115.21, 116.35)	76.55 ± 0.22 (76.12, 76.98)
	M	98.12 ± 0.63 (96.89, 99.36)	150.65 ± 3.74 (143.32, 157.98)**	50.47 ± 0.31 (49.87, 51.08)**	83.43 ± 0.13 (83.17, 83.69)**	119.16 ± 0.38 (118.41, 119.90)	79.67 ± 0.30 (79.08, 80.27)
	F	93.45 ± 0.56 (92.36, 94.55)	107.96 ± 4.32 (99.48, 116.44)	57.01 ± 0.36 (56.30, 57.71)**	76.43 ± 0.13 (76.18, 76.69)**	112.33 ± 0.39 (111.57, 113.08)	73.45 ± 0.26 (72.94, 73.95)
*P* for trend	Total	<0.005	<0.005	<0.001	<0.001	0.499	0.458
	M	0.73	0.023	<0.001	<0.001	0.78	0.38
	F	<0.005	0.045	<0.001	<0.001	0.75	0.80

All variables adjusted for age, sex, and BMI*. P* trend is from Wald test

*P* < 0.001 for differences from 0/week

* <0.05, ** <0.001

### Association between prevalence of metabolic syndrome and physical activity according to type and frequency of physical activity

The association between the frequency of physical activity for the five different types of physical activity and the prevalence of metabolic syndrome is presented in Table 3. The proportion of metabolic syndrome was significantly lower among participants who participated in moderate physical activity, walking, and flexibility compared with subjects who did not participate in those three physical activity. The frequency of physical activity was found to be associated with the prevalence of metabolic syndrome for vigorous physical activity, moderate physical activity, walking, strength, and flexibility.

**Table 3:** The odds ratio for metabolic syndrome according to physical activity type and frequency

Physical activity	Frequency (time/week)	Unadjusted	Adjusted		
		OR (95% CI)	OR (95% CI)	Sex	OR (95% CI)
Vigorous	0/week	1.00	1.00	M	1.00
				F	1.00
	1/week	0.93 (0.81–1.05)	0.92 (0.80–1.07)	M	0.92 (0.78–1.09)
				F	0.90 (0.69–1.18)
	2/week	1.10 (0.95–0.1.25)	0.97 (0.82–1.14)	M	0.97 (0.80–1.19)
				F	0.96 (0.72–1.13)
	3/week	0.95 (0.81–1.1)	*0.81* (*0.67*–*0.97*)	M	0.81 (0.64–1.02)
				F	0.82 (0.51–1.11)
	4/week	0.93 (0.73–1.17)	0.87 (0.66–1.14)	M	0.79 (0.56–1.11)
				F	1.08 (0.69–1.19)
	5/week	0.82 (0.65–1.05)	*0.71*(*0.54*–0*.94*)	M	0.80 (0.58–1.01)
				F	*0.54* (*0.33*–*0.90*)
	6/week	0.84 (0.61–1.15)	*0.65*(*0.45*–*0.94*)	M	0.67 (0.43–1.03)
				F	0.60 (32–1.12)
	Everyday	0.91 (0.69–1.20)	*0.64* (*0.46–0.88*)	M	*0.58* (*0.39–0.87*)
				F	0.76 (0.46–1.13)
Moderate	0/week	1.00	1.00	M	1.00
				F	1.00
	1/week	*0.80* (*0.70–0.92*)	*0.80* (*0.69–0.93*)	M	77 (0.64–93)
				F	0.84 (0.64–1.10)
	2/week	0.93 (0.80–1.07)	0.87 (0.74–1.03)	M	0.83 (0.68–1.12)
				F	*0.95* (*0.73–1.13*)
	3/week	0.92 (0.80–1.07)	0.90 (0.76–1.08)	M	0.89 (71–1.12)
				F	0.92 (0.72–1.19)
	4/week	1.04 (0.85–1.28)	0.87 (0.68–1.10)	M	0.84 (0.61–1.16)
				F	0.89 (0.62–1.30)
	5/week	*0.72* (*0.58–0.89*)	*0.68* (*0.54–0.87*)	M	0.76 (0.57–1.03)
				F	*0.54* (*0.36–0.81*)
	6/week	0.84 (0.65–1.09)	0.76 (0.54–1.01)	M	0.57 (0.39–0.82)
				F	1.30 (0.82–2.04)
	Everyday	1.04 (0.88–1.23)	0.90 (0.74–1.1)	M	*0.79* (*0.60–1.03*)
				F	1.05 (0.81–1.34)
				F	1.00
Walking	0/week	1.00	1.00	M	1.00
				F	1.0
	1/week	0.92 (0.77–1.10)	1.1 (0.90–1.35)	M	1.18 (0.91–1.52)
				F	0.95 (0.69–1.31)
	2/week	0.88 (0.75–0.1.02)	0.92 (0.76–0.1.1)	M	1.00 (0.78–1.27)
				F	0.80 (0.61–1.06)
	3/week	*0.84* (*0.73–0.97*)	0.90 (0.76–1.1)	M	0.94 (0.73–1.21)
				F	0.86 (0.68–1.11)
	4/week	0.84 (0.70–1.00)	0.90 (0.73–1.12)	M	0.86 (0.64–1.15)
				F	0.98 (0.72–1.33)
	5/week	*0.67* (*0.56–0.79*)	0.87 (0.71–1.11)	M	0.95 (73–1.24)
				F	0.77 (0.59–1.01)
	6/week	*0.58* (*0.48–0.71*)	*0.67* (*0.53–0.85*)	M	*0.56* (*0.41–0.77*)
				F	0.89 (0.63–1.24)
	Everyday	*0.76* (*0.67–0.86*)	0.88 (0.76–1.02)	M	0.86 (0.70–1.05)
				F	0.92 (0.74–1.14)
Strength	0/week	1.00	1.00	M	1.00
				F	1.00
	1/week	0.95 (0.81–1.11)	0.93 (0.77–1.11)	M	0.96 (0.78–1.12)
				F	0.81 (0.58–1.13)
	2/week	0.92 (0.79–0.1.07)	0.84 (0.71–0.1.00)	M	0.84 (0.69–1.04)
				F	0.84 (0.59–1.21)
	3/week	0.92 (0.77–1.09)	*0.75* (*0.62–0.92*)	M	*0.78 (0.72*–*0.98)*
				F	*0.69 (0.46*–*1.02)*
	4/week	0.90 (0.72–0.1.12)	*0.70* (*0.54–0.90*)	M	*0.69 (0.52*–*0.92)*
				F	*0.74 (0.47*–*1.17)*
	More than 5/week	1.01 (0.86–1.20)	*0.75* (*0.62–0.90*)	M	*0.75 (0.61*–*0.92)*
				F	*0.77 (0.53*–*1.10)*
Flexibility	0/week	1.00	1.00	M	1.00
				F	1.00
	1/week	*0.81* (*0.70–0.92*)	0.93 (0.78–1.09)	M	0.95 (0.76–1.17)
				F	0.89 (0.69–0.1.13)
	2/week	*0.83* (*0.74–0.94*)	0.91 (0.79–0.1.04)	M	0.91 (0.76–1.10)
				F	0.91 (0.73–1.14)
	3/week	*0.82* (*0.72–0.94*)	*0.76* (*0.65–0.88*)	M	*0.77* (*0.63–0.93*)
				F	*0.76* (*0.61–0.94)*
	4/week	0.89 (0.74–1.06)	*0.79* (*0.65–0.97*)	M	*0.74* (*0.57–0.96*)
				F	0.92 (0.69–1.22)
	More than 5/week	0.94 (0.84–1.05)	*0.78* (*0.68–0.90*)	M	*0.76 (0.64–0.90)*
				F	0.84 (0.67–1.04)

*OR* odd ratio, *CI* confidence interval, Multivariable logistic regression adjusted for age, sex, and Body Mass Index (BMI)

The lowest prevalence of metabolic syndrome was observed in those participants who walked six times per week (OR 0.67, 95% CI 0.53–0.85) as adjusted for confounders (age, sex and BMI), and compared with participants who did not do any walking or other physical activity. The beneficial effects of physical activity on metabolic syndrome varied widely based on type and frequency of physical activity.

#### Additional analysis between sexes

We found that the association between physical activity and prevalence of metabolic syndrome was similar between the sexes, except for triglycerides. The average triglyceride level for men (158.73 ± 1.59 mg/dL) was higher than for women (106.74 ± 0.98 mg/dL) at baseline. The men who participated in more than 4/week of vigorous exercise, moderate exercise, and strength exercise and more than 6/week of walking had lower than 150 mg/dL of triglycerides. The average triglyceride level in men who did not participate in vigorous exercise was 159.14 ± 1.88 mg/dL, but for men who participated in vigorous exercise every day the average triglyceride level was 133.07 ± 7.35 mg/dL. The results showed no significant differences after adjusting hormonal status.

## Discussion

It is well known that physical activity has favorable effects toward preventing metabolic syndrome. This study investigated not only the association between risk components of metabolic syndrome and physical activity type and frequency, but also examined the association between the prevalence of metabolic syndrome and physical activity according to type and frequency of physical activity.

The main finding of this study is that physical activity reduces the prevalence of metabolic syndrome. The lowest prevalence of metabolic syndrome was investigated in those participants who walked six times per week (OR 0.67, 95% CI 0.53–0.85). Several studies have previously determined the association between physical activity and the prevalence of metabolic syndrome, by examining the amount of physical activity during a physical activity (Laaksonen et al. 2002; Ekelund et al. 2005). The added value of this study is that it provides detailed support for that relationship, and also provides a more specific examination of the frequency and type of physical activity. Laaksonen et al. (2002) investigated the association between time spent (>3 h/week) doing moderate and vigorous physical activity and the prevalence of metabolic syndrome, as measured by self-reported data. Similar to this study, participants who participated in vigorous physical activity, and were in the upper third of the VO_2max_ range, had an even stronger inverse association with the occurrence of metabolic syndrome. Ekelund et al. (2005) found a dose–response association between physical activity energy expenditure, including all daily activity, regardless of physical activity type and the metabolic syndrome. Whereas, in this study the finding was a dose–response association between physical activity and the prevalence of metabolic syndrome, and provided detailed supporting analyses covering main five-physical activity types.

Another main finding was that the measured levels of risk components of metabolic syndrome improved as subjects participated in vigorous physical activity, moderate physical activity, walking, strength, and flexibility, when compared with subjects who did not participant in physical activity. As the frequency of physical activity increased, the level of risk components of metabolic syndrome decreased in walking with fasting blood glucose, moderate physical activity with HDL cholesterol, and vigorous physical activity, strength, and flexibility with waist circumferences. In general, physical activity can be considered an effective prevention and treatment for metabolic syndrome, noting that each component of metabolic syndrome is impacted uniquely depending on the specific type and frequency of physical activity. As a guideline, vigorous physical activity at least once per week and regular physical activity of moderate, walking, strength, and flexibility for more than three times a week might be recommended for reducing the prevalence of metabolic syndrome.

All of the five types of physical activity had favorable effects toward increasing HDL cholesterol and reducing triglycerides. Varying the type and frequency of physical activity gives rise to different results as indicated in previous studies (Andersen et al. 1999; Johnson et al. 2007; Houmard et al. 2004; Gennuso et al. 2014). Generally, moderate to vigorous exercise was effective towards reducing triglyceride and body mass (Andersen et al. 1999). Jonson et al. (2007) provided additional support as to the amount and intensity of exercise needed to significantly improve factors related to metabolic syndrome and recommended that 30 min of moderate intensity exercise be participated in every day, when the objective is to reduce the prevalence of metabolic syndrome. Similarly, Houmard et al. (2004) reported that moderate intensity exercise resulted in better triglyceride responses and insulin sensitivity, when compared with vigorous intensity exercise. Gennuso et al. (2014) found that increased amounts of sedentary time were associated with an increased risk of metabolic syndrome, including triacylglycerol, HDL-cholesterol, and fasting glucose. Also, this study found favorable effects on fasting blood glucose from even walking once per week, in addition to everyday walking and vigorous physical activity. Aerobic capacity, musculoskeletal, and hemodynamic improvements were achieved by greater duration and intensity or physical activity, and even a relative low frequency of physical activity (10 min walking three times per week) improved fasting plasma glucose (Yates et al. 2013; Foulds et al. 2014).

An interesting finding from this study was that waist circumferences decreased for all types of physical activity, however waist circumferences were not decreased with walking (for once per week to four times per week). The indication is that walking may be too low in intensity to reduce the waist circumference. In a similar study, Chang et al. (2015) measured waist circumferences of 628 senior adults, in order to investigate a combined association among body compositions, metabolic syndrome factors, and physical fitness characteristics and found that the occurrence of metabolic syndrome was positively associated with reduced flexibility (OR 0.9, 95% CI 1.25–2.07). Additionally, a finding of this study was that participants who did flexibility physical activity three times a week, had a decreased occurrence of metabolic syndrome (OR 0.76, 95% CI 0.65–0.88). We would suggest that flexibility should be an indicator of evaluation for metabolic syndrome and found that increased physical fitness levels caused other metabolic risk factors to decrease.

Physical activity helps that these metabolic factors were systolic blood pressure, when participating in walking and strength at least two times per week; and diastolic blood pressure during vigorous physical activity, when participated in only once per week or three times per week. For other types and frequency of physical activity, this study could not find significant changes. Although blood pressure in this study did not find similar significant correlations in examining other components of metabolic syndrome, other previous study did hold that physical activity was strongly recommended for patients who had atherosclerotic cardiovascular disease, in order to better manage blood pressure due to resultant improved lipoprotein profiles, including LDL-cholesterol and HDL-cholesterol (Smith et al. 2009; Sang et al. 2014).

Additional analysis was conducted to find differences in the relationship between exercise and metabolic syndrome based on sex. The result showed that there was a similar association between physical activity and prevalence of metabolic syndrome. However, a major difference was found in the triglyceride levels. Women had about a 50 mg/dL lower triglyceride level than men at baseline. The men who did not participate in exercise had over 150 mg/dL, which is a borderline for metabolic syndrome. On the other hand, the men who participated in vigorous exercise, moderate exercise and strength exercise over 4 times per week and walking for over 6 times per week were found to be under the triglyceride borderline level. Similarly, Wood et al. (1991) reported that a 12 month exercise intervention program with dietary modifications reduced triglyceride levels by 30% in men and 2% in women. Also, Brownell et al. (1982) found higher beneficial effects of exercise (after a 10 week program) on triglyceride in men than for women. We confirmed that Korean men might receive a higher benefit from participation in physical activity towards amending the triglyceride level than women, among the metabolic syndrome components.

The mechanism behind the effects of physical activity on metabolic syndrome might be related to a resultant reduction in the level of inflammation grade. Although intense physical activity invokes pro-inflammatory cytokines, a possible explanatory mechanism is that physical activity improves body composition, dyslipidemia and endothelial function, increases anti-inflammatory cytokines, decreases body fat, and decreases the expression of adhesion molecules (Powers and Hamilton 1999; Green et al. 2004; Kasapis and Thompson 2005).

The strength of this study is that it identifies the healthier frequencies of physical activity and distinguishes which type of physical activity should be conducted to better reduce the risk of metabolic syndrome. The study findings serve to provide key guidance for reversing the prevalence of metabolic syndrome based on type and frequency of physical activity, by employing a large representative sample. In this way the study was able to disentangle the effects of physical activity in five different types of physical activity, while looking across various frequencies of physical activity. The strength of the sampling methodology is supported by the use of KHANES data that is a valid and reliable countrywide database. The data analysis approach used statistical analyses that employed a stratified multistage sampling design.

There are several study limitations that need to be addressed. First, the type and frequency of physical activity in this study were measured by using a self-reporting style IPAQ survey instrument, and it is noted that self-reported data could lead to misclassification and measurement error. To address that weakness, a trained investigator conducted the survey, and this approach has been validated in previous studies. Second, this study does not infer causality between type and frequency of physical activity and the prevalence of metabolic syndrome, because this study used a cross-sectional design. Third, the Korean National Health and Nutrition Examination Survey did not provide detailed data related to the use of specific medications. As such, this study was unable to identify the relationships associated with those factors. Fourth, An important idea to grasp is that if a study is very large, its result may be statistically significant, however, that level of deviation from the null hypothesis may be too small to be of any clinical interest. That is, the statistical difference is too small to warrant a change or modification to current clinical practices or to the current standardized treatment programs. Lastly, physical activity intensity, frequency, and type should be more detailed in order to explain the exact mechanisms and associations between physical activity and metabolic syndrome.

In conclusion, physical activity was found to be associated with the components of metabolic syndrome and with the prevalence of metabolic syndrome. It was found that the effects of physical activity varied significantly depending on the type and frequency of the physical activity participation. Regular physical activity and the avoidance of physical inactivity are key principles for the prevention of metabolic syndrome. When patients are diagnosed with metabolic syndrome, it is important to check the underlying risk components related to metabolic syndrome. From that information, better recommendations can be made that address specific physical activity types and frequencies that could benefit the patient in their efforts to reverse the condition of metabolic syndrome.

## Additional file

**Additional file 1: Table S1.** Modified international physical activity questionnaire.
