# Supplementary material for: Association between physical activity and the prevalence of metabolic syndrome: from the Korean National Health and Nutrition Examination Survey, 1999–2012
Source: Springerplus. 2016 Oct 25;5(1):1870. doi: 10.1186/s40064-016-3514-5 (PMC5080278; doi:10.1186/s40064-016-3514-5)
Supplement: Supplementary file 1 — Additional file 1: Table S1. Modified international physical activity questionnaire. [file 40064_2016_3514_MOESM1_ESM.docx]

**MODIFIED INTERNATIONAL PHYSICAL ACTIVITY QUESTIONNAIRE**

| 1. During the last 7 days, on how many days did you do vigorous physical activities like heavy lifting, digging, aerobics, or fast bicycling?  1) I did not do any physical activity  2) one day per week  3) two days per week  4) three days per week  5) four days per week  6) five days per week  7) six days per week  8) every day |
| --- |
| 2. During the last 7 days, on how many days did you do moderate physical activities like carrying light loads, bicycling at a regular pace, or doubles tennis? Do not include walking.  1) I did not do any physical activity  2) one day per week  3) two days per week  4) three days per week  5) four days per week  6) five days per week  7) six days per week  8) every day |
| 3. During the last 7 days, on how many days did you walk for at least 10 minutes at a time?  1) I did not do any physical activity  2) one day per week  3) two days per week  4) three days per week  5) four days per week  6) five days per week  7) six days per week  8) every day |
| 4. During the last 7 days, on how many days did you do strengthening physical activity like resistance training and weight training?  1) I did not do any physical activity  2) one day per week  3) two days per week  4) three days per week  5) four days per week  6) more than five days per week |
| 5. During the last 7 days, on how many days did you do flexibility physical activity like stretching?  1) I did not do any physical activity  2) one day per week  3) two days per week  4) three days per week  5) four days per week  6) more than five days per week |
